# The effect of *Elettaria cardamomum* (cardamom) on the metabolic syndrome: Narrative review

**DOI:** 10.22038/IJBMS.2021.54417.12228

**Published:** 2021-11

**Authors:** Roghayeh Yahyazadeh, Mahboobeh Ghasemzadeh Rahbardar, Bibi Marjan Razavi, Gholamreza Karimi, Hossein Hosseinzadeh

**Affiliations:** 1 Department of Pharmacodynamics and Toxicology, School of Pharmacy, Mashhad University of Medical Sciences, Mashhad, Iran; 2 Pharmaceutical Research Center, Pharmaceutical Technology Institute, Mashhad University of Medical Sciences, Mashhad, Iran; 3 Targeted Drug Delivery Research Center, Pharmaceutical Technology Institute, Mashhad University of Medical Sciences, Mashhad, Iran

**Keywords:** Anti-inflammatory agents, Anti-oxidants, Elettaria cardamomum, Hypoglycemic agents, Hypolipidemic agents, Metabolic syndrome

## Abstract

Metabolic syndrome (MetS), as a health-threatening factor, consists of various symptoms including insulin resistance, high blood sugar, hypertension, dyslipidemia, inflammation, and abdominal obesity that raise the risk of diabetes mellitus and cardiovascular disease. Cardiovascular diseases are important causes of mortality among the world population. Recently, there has been a growing interest in using phytomedicine and natural compounds in the prevention and treatment of various diseases. The data was gathered by searching various standard electronic databases (Google Scholar, Scopus, Web of Science, and PubMed) for English articles with no time limitations. All *in vivo*, *in vitro*, and clinical studies were included. *Elettaria cardamomum* (cardamom) is a rich source of phenolic compounds, volatile oils, and fixed oils. Cardamom and its pharmacologically effective substances have shown broad-spectrum activities including antihypertensive, anti-oxidant, lipid-modifying, anti-inflammatory, anti-atherosclerotic, anti-thrombotic, hepatoprotective, hypocholesterolemic, anti-obesity, and antidiabetic effects. This review aims to highlight the therapeutic effects of cardamom on MetS and its components including diabetes, hyperlipidemia, obesity, and high blood pressure as well as the underlying mechanisms in the management of MetS. Finally, it can be stated that cardamom has beneficial effects on the treatment of MetS and its complications.

## Introduction

Metabolic syndrome (MetS), as a metabolic disorder, includes a set of major symptoms such as impaired glucose level, dyslipidemia, central obesity, overweight, insulin resistance, and high blood pressure that lead to cardiovascular diseases (CVD), cancer, diabetes mellitus type 2 (DMT2), short lifespan, and low-quality life. MetS has increased concerns about public health problems throughout the world population (1). It is mentioned as a leading public health problem and the cause of morbidity and mortality via high risks for increasing diabetes mellitus and CVD (2-4). Pathophysiology of MetS -induced complications is possibly due to an imbalance in calorie and energy intake associated with alterations in genetic and lifestyle. In addition, it may be affected by the type of food and gut microorganisms (5). Gut microorganisms are sensitive to the changes in nutrition in the intestine and they can also directly participate in the process of absorption (6).

The prevalence of the MetS is not the same in different countries such as the USA (34%), India (25.6%), Kuwait (24.8%), and Australia (22.1%) (7-10). MetS is a threat to human health that requires vital prevention and treatment; thus, effective strategies must be applied to decrease the burden of this disease (11). Because of the side effects and inefficiency of drugs, herbal medicines have been considered for their potential impact on improving and maintaining human health. Medicinal plants and their active constituents such as grapes***, ***saffron***,*** rosemary, garlic, and rutin have long been used to treat various disorders (3, 12-15). Generally, the purpose of spices using in food is to increase the flavor and to take advantage of their medicinal properties (16). Many of the spices belong to herbal families. Therefore, the demand for these herbs has increased because of their beneficial effects on various disorders. Herbal medicines such as *Vitis vinifera* (15), *Silybum marianum* L. (17),* Nigella sativa* (18, 19), *Allium sativum* (12), *Persea americana* (20), *Solanum melongena *(21), and *Berberis vulgaris* (22) can ameliorate MetS.  

**Figure 1:**
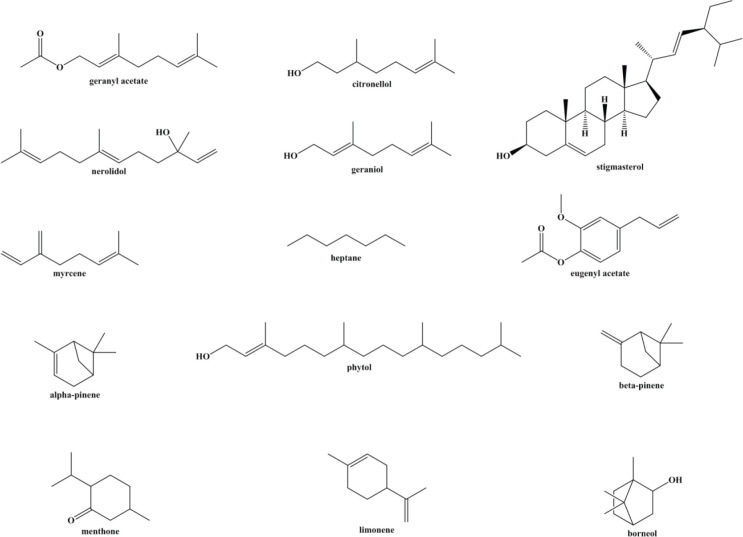
Active ingredients of cardamom

*Elettaria cardamomum *also known as cardamon, belongs to the Zingiberaceae family. It is native to the Indonesia and Indian subcontinent, Pakistan, Burma, Bangladesh, tropical and subtropical Asia. It is perennial, herbaceous monocots with 4-5 m in height. Cardamom flowers are the whitish lip located at the tip of the corolla tube (23). Phytochemical investigations illustrated that *E. cardamomum *contains terpinene, stigmasterol, geranyl acetate, geraniol, β-pinene, citronellol, borneol, bisabolene, eugenyl acetate, phytol, β-sitostenone, nerolidol, linalol, α-pinene, menthone, cineol, limonene, subinene, heptane, myrcene, and α-terpineol (24, 25) (Figure 1). The small seeds of cardamom have a triangular pod in cross-section that is covered by a thin papery black shell (26). Several studies have reported the beneficial effects of cardamom on Gram-positive and Gram-negative bacteria (27, 28), teeth and gums health (29), lung disorders (30), and gastrointestinal disorders (31, 32). The therapeutic impacts of cardamom are due to its pharmacological effects including anti-oxidant, antimutagenic (33), antibacterial, anti-inflammatory (34), antidiabetic (35), cardioprotective (36), hepatoprotective, and chemoprotective properties (37).

This review summarizes the potential efficacy of cardamom and its active constituent in MetS and its related complications. 

**Methods**

 PubMed, Web of Science, Google Scholar, and Scopus databases were used to find articles from 1995 to 2021. The search terms used were “cardamom”, “*Elettaria cardamomum*”, “hypertension”, “diabetes”, “hyperglycemia”, “antihyperglycemic”, “antidiabetic”, “blood glucose”, “dyslipidemia”, “hyperlipidemia”, “hypercholesterolemia”, “hyper-triglyceridemia”, “atherosclerosis”, “obesity”, “appetite”, “anti-obesity”, “weight loss”, and “metabolic syndrome”. Our team collected all published *in vivo*, *in vitro *articles, and clinical studies investigating the ameliorative effects of cardamom and its effective constituents on MetS. 

**Effect of** ***E. cardamomum*** **on dyslipidemia**

Dyslipidemia, which is linked to a changed lipoprotein spectrum and modified lipoproteins, is one of the main risk factors in MetS (38). Increased triglyceride (TG)-rich lipoproteins (TRLs), reduced high-density lipoprotein (HDL) and augmented small low-density lipoprotein (LDL) particles are the three major components of dyslipidemia associated with MetS (39). Dyslipidemia plays an important role in developing atherosclerotic CVD associated with MetS. The association between low-density lipoprotein cholesterol (LDL-C) levels and the initiation and development of arterial plaques, for example, is well known, and LDL-lowering therapy has been shown in several clinical trials to substantially reduce the frequency of cardiovascular events. Furthermore, epidemiologic studies have shown that high-density lipoprotein cholesterol (HDL-C) levels and coronary artery disease have a clear inverse relationship (40). Some studies have also stated that the C-reactive protein (CRP) level is a marker for dyslipidemia, diabetes, and MetS (41, 42). 

Cardamom and its active ingredients have been demonstrated to modify blood total cholesterol (TC), TG, LDL, and HDL in several investigations.

***Clinical trials***

A clinical trial was carried out on obese or overweight pre-diabetic women taking 3 grams of cardamom for 2 months. The obtained results disclosed that mean TC (from 192.6 to 183.7 mg/dl) and LDL-C (from 118.1 to 110.5 mg/dl) significantly reduced. It also showed a protective effect on HDL-C (from 44.1 to 42.7 mg/dl) amount in pre-diabetic subjects (43). Another study reported that cardamom supplementation (3 g, 10 weeks) could significantly decrease TG (from 158.4 to 125.8 mg/dl) in T2DM patients in comparison with the placebo group (44). A study aimed to assess the cardamom (3 g/day, 8 weeks) effects on inflammation and oxidative stress in hyperlipidemic pre-diabetic women. In comparison to the placebo group, cardamom supplementation significantly reduced serum hs-CRP (from 5.2 to 5.06 mg/dl), hs-CRP/IL-6 ratio (from 775.04 to 623.5), and MDA (from 8.7 to 7.3 μM) level. Cardamom has been shown to control certain inflammatory and oxidative stress parameters in pre-diabetic people. As a result, it may help these patients avoid complications related to inflammation and oxidative stress (45).

***In vivo*** **studies**

Pre-clinical research reported that the administration of cardamom powder suspension in 2% gum acacia (1 g/kg/10 ml, oral, for 12 days) decreased the levels of TC and TG, and increased HDL levels in rats with dexamethasone-induced hepatic steatosis (46). In an animal study, cardamom oil (3 g/kg, equivalent to 50 g/kg cardamom, 8 weeks, PO) is effective in restoring lipid homeostasis in hypercholesterolemia rats. The substantial reduction in atherogenicity index following dietary intervention with cardamom powder and oil suggests that cardamom may have a cardioprotective effect (47). 

***In vitro*** **studies**

 An *in vitro* study indicated that 1,8-cineole, a constituent of cardamom, exhibited anti-oxidant properties in lipoprotein metabolism and reduced lipid accumulation in THP-1 cells (48). 

Cardamom may exhibit its anti-hyperlipidemic effects through the enhanced rate of cholesterol degradative processes or lipoprotein lipase activity, as well as the efficient reduction in lipid absorption from the intestine (Table 1).

**Effect of** ***E. cardamomum*** **on obesity**

Obesity is a medical condition in which extra body fat has stored to the point that it can be harmful to one’s health. It is characterized by BMI, which is a metric for measuring body fatness (49, 50). Obesity might result in several diseases, including DMT2 (51), cardiovascular disease (52), hypertension (53), as well as respiratory disease (54). Oxidative stress and inflammation have important roles in metabolic disorders linked to obesity (55, 56). According to previous reports, obese patients have lower levels of anti-oxidant markers (superoxide dismutase (SOD), glutathione (GSH) in their blood (55, 57, 58).

Moreover, several parts of the hypothalamus are implicated in the development and maintenance of obesity through several pathways. As one of these important metabolic regulators, sirtuins (SIRTs) are a well-conserved family of class III deacetylases (59). In mammals, seven members of the SIRT family (SIRT1-7) have been identified. SIRT1 is expressed in different organs including the pancreas, liver, adipose tissue, muscle, heart, as well as important metabolic centers of the brain such as the ventromedial hypothalamus (VMH), dorsomedial nucleus, and paraventricular nucleus of the hypothalamus (PVN) (60). SIRT1 regulates body weight by controlling metabolic processes such as food intake, adiposity, energy expenditure, thermogenesis of brown adipose tissue, and browning of white adipose tissue (61, 62). A high-fat diet and obesity result in downregulation of sirtuins especially SIRT1 expression in humans (63). SIRT1 regulates systemic homeostasis by preventing Forkhead box protein O1 (FoxO1) acetylation. In the insulin signaling pathway, FoxO1 is a downstream transcription factor and its activation or overexpression in the hypothalamus decreases insulin anorexigenic property (64), increases adiposity, and results in weight gain (65).

The anti-obesity effects of cardamom and its active constituents have been stated in numerous studies (Table 2).

**Clinical trials**

An investigation suggested that the administration of cardamom (3 g/day, 3 months) caused an increase in SIRT1 (from 1.2 to 1.3 ng/ml) in the non-alcoholic fatty liver patient (66). Another clinical trial explained that administration of green cardamom (3 g/day, 16 weeks) controlled the expression of some diabetes and obesity genes including fat mass and obesity-associated (FTO), carnitine palmitoyltransferase 1A (CPT1A), leptin receptor (LEPR), lamin A/C, and peroxisome proliferator-activated receptor gamma (PPAR-γ) in women with polycystic ovary syndrome (67).

***In vivo*** **studies**

It was also found that administration of cardamom powder (1% of powder chow diet w/w, 8 weeks) prevented obesity in high-fat diet-induced obese rats (16).

**Effect of** ***E. cardamomum*** **on hypertension**

Hypertension is a metabolic risk factor for CVD. It raises the possibility of numerous CVDs, such as peripheral vascular disease, heart failure, coronary artery disease, and stroke (18). 

Several lines of evidence have revealed that cardamom and its active constituents show hypotensive and cardiovascular protective properties.

**Clinical trials**

A study showed that treatment with cardamom (3 g/day, for 12 weeks) caused a significant decrease in systolic blood pressure (SBP) (from 154.2 to 134.8 mmHg) and diastolic blood pressure (DBP) (from 91.8 to 79.6 mmHg) in individuals with primary hypertension of stage 1. Also, cardamom ameliorated ﬁbrinolysis and improved anti-oxidant status after 3 months (68). In contrast, it has been reported that the administration of cardamom (3 g/day, 8 weeks) did not significantly reduce SBP (from 120.7 to 115.5 mmHg) and DBP (from 78.1 to 77.5 mmHg) in obese pre-diabetic women which might be because of a short intervention period and a small sample size (43).

***In vivo*** **studies**

An *in vivo* study showed that cardamom crude extract (3-100 mg/kg) reduced blood pressure by a combination of cholinomimetic and calcium channel blocking mechanisms in rats (69). 

It has been reported that α-terpineol (25, 50, or 100 mg/kg/day, a week, PO) was able to attenuate mean arterial pressure in hypertensive rats. The authors suggested that α-terpineol can decrease arterial pressure, Probably by decreasing vascular resistance and repairing enzymatic anti-oxidants in these animals (70). These findings reinforce the results of another research that demonstrated that α-terpineol (1, 5, 10, 20 and 30 mg/kg, IV) could induce hypotension and vasorelaxation mediated, partly by the endothelium, probably through releasing NO and activating the NO–cGMP pathway (71). The IV administration of 1,8-cineole (0.3–10 mg/kg) to rats reduced blood pressure, most likely due to active vascular stimulation rather than a reduction of sympathetic tone. These results propose that 1,8-cineole helps to mediate the hypotensive effects of essential oils from certain aromatic plants that are commonly used to treat hypertension (72). 

***In vitro*** **studies**

The vasodilatory effect of cardamom (3-10 mg/ml) has been shown against contraction induced by phenylephrine (1 µM) and potassium (80 mM) in the isolated rat aorta (69). An *in vitro* study on rabbit aortic endothelial cell line revealed that α-terpineol (10^−6^, 10^−5^, and 10^−4^ mol/l for 15 min) increased nitric oxide (NO) levels via NO–cGMP pathway activation (71). 

**Figure 2:**
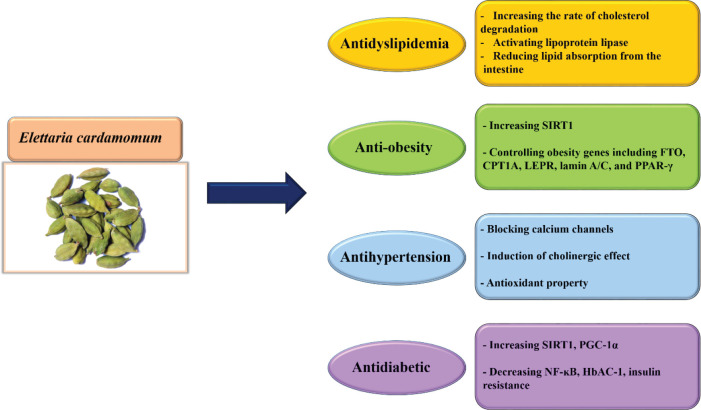
Schematic effect of cardamom and its active component on metabolic syndrome

As a result, different mechanisms such as calcium channel blocking, induction of NOS and endothelial NO, cholinergic effect, and anti-oxidant property are included in the cardioprotective and hypotensive effects of cardamom and its active ingredients (Figure 2). Based on these studies, cardamom is a high potential cardiovascular protective agent (Table 3).

**Effect of** ***E. cardamomum*** **on hyperglycemia**

Hyperglycemia is one of the risk factors of metabolic syndrome. Hyperglycemia can induce vascular inflammation (73), microvascular damage (74), and atherosclerosis (75). It also impairs the immune status by stimulating cell adhesion molecules and inflammatory cytokines besides inhibiting the function of leukocytes (76). 

Cardamom can ameliorate high blood glucose, insulin resistance, and glucose metabolic disorders. *E. cardamomum *and its active constituents can control insulin secretion, insulin resistance through increasing the amount of SIRT1, PPAR-γ coactivator-1 alpha (PGC-1α), and attenuating the function of nuclear factor kappa-light-chain-enhancer of activated B cells (NF-κB) as well as controlling glucose metabolism by inhibiting α-glucosidase and α-amylase.

Relevant studies regarding the effect of cardamom on diabetes, insulin resistance, and glucose metabolism will be discussed below.

**Clinical trials**

A clinical trial demonstrated that administration of cardamom (3 g/day, 3 months) to a non-alcoholic fatty liver patient caused an increase in SIRT1 (from 1.2 to 1.3 ng/ml) (66). SIRT1 is responsible to regulate insulin secretion, insulin resistance, lipid/glucose/energy metabolism, inflammatory process, CVD, and kidney diseases (77). Moreover, SIRT1 can upregulate PGC-1α that inhibits NF-κB activation. It also impacts obesity, hepatic glucose production, insulin sensitivity (78), inhibits oxidative stress, and inflammation in pancreatic β-cells (79). On the other hand, NF-κB activation in adipose tissue macrophage of liver and muscle adipose tissue can contribute to the development of insulin resistance in these tissues (80). The administration of cardamom (3 g/day, 10 weeks) declined serum hemoglobin-A1C (HbA1C) (from 8.19 to 7.71 %), homeostatic model assessment-insulin resistance (HOMA-IR) (from 5.01 to 3.80), insulin (from 12.8 to 10.7 μIU/dl), TG levels (from 158.4 to 125.8 mg/dl), and elevated SIRT1 level (from 8.73 to 11.10 ng/dl) in overweight/obese T2DM patients (44). It was also observed that cardamom (3 g, 2 months) could increase insulin sensitivity (from 0.30 to 0.31 QUICKI) in pre-diabetic subjects (43). 

***In vivo*** **studies**

It has been shown that oral administration of cardamom suspension (1 g/kg/10 ml of 2% gum acacia, 6 days before dexamethasone and 6 days during dexamethasone administration (to induce hyperglycemia, dyslipidemia, and hepatic steatosis)) reduced hepatomegaly and hyperglycemia in Albino rats (46). Nanoliposome of 1,8-cineole rich extract of cardamom (550 mg/kg, 35 days) could manage T2DM and hypercholesterolemia by decreasing fasting blood sugar and controlling serum lipid profile (81). It was demonstrated that cardamom powder (1% of powder chow diet w/w, 8 weeks) could significantly reduce glucose intolerance, oxidative stress, and inflammation in the liver of obese rats fed with a high carbohydrate high-fat diet (16). 

Moreover, it was reported that supplementation of *E. cardamomum* extract (100, 200, and 400 mg/kg) for 8 weeks in diabetic rats reduced insulin resistance in animals’ brains by increasing glutamate receptor expression (α-amino-3-hydroxy-5-methyl-4-isoxazolepropionic acid (AMPA) glutamate receptor 1 (GluR1) subunit and N-methyl-D-aspartate receptor (NMDA) receptor subunits NR1, NR2A, NR2B) (82).

***In vitro*** **studies**

It has been reported that supercritical carbon dioxide extract of cardamom increased both the liver insulin sensitivity and glucose uptake in the gut. The results of this study showed that this extract of spices is a safe alternative to metformin and blood glucose regulator-34m (BGR-34) in the treatment of T2DM and could be tested in clinical trials (83). The hydrolysis of starch by pancreatic α-amylase and the uptake of glucose by intestinal α-glucosidases generate a quick spike in blood glucose levels, which causes hyperglycemia in T2DM patients (84). Therefore, an effective strategy to manage DM is inhibiting intestinal α-glucosidase and α-amylase. An *in vitro* study on aqueous and methanolic cardamom extracts showed that these extracts have α-glucosidase and α-amylase inhibitory effects and can be used in managing diabetes (35).

It can be suggested that cardamom and its active ingredients disclose their therapeutic or preventive properties against diabetes via several mechanisms including decreasing glucose level, insulin resistance, increasing insulin level, glucose uptake, anti-oxidant eﬀects, and the number of β-cells in the pancreas (Table 4) (Figure 2). However, there are not enough clinical studies on the effectiveness of cardamom in diabetes. Therefore, more human studies should be performed on the preventive or curative effect of this herb on diabetes. 

**Table 1:** Therapeutic effects of cardamom and its active constituents on dyslipidemia

**Compounds**	**Dose**	**Study design**	**Results**	**Ref**
Cardamom	3g/day, 2 months	Clinical trial*, * pre-diabetic women	↓ TC ↓ LDL-C ↓ Insulin sensitivity	(43)
Cardamom	3g/day, 10 weeks	Clinical trial, T2DM patients	↓ HbA1C ↓ insulin ↓ HOMA-IR ↓ TG ↑ SIRT1	(44)
Cardamom	3g/day, 8 weeks	Clinical trial, hyperlipidemic, overweight, and obese pre-diabetic women	↓ hs-CRP ↓ hs-CRP/IL-6 ↓ MDA	(45)
Cardamom suspension of 2% gum acacia	1 g/kg/10 mL, p.o.	*In vivo*, albino rats	↓ Hepatomegaly ↓ Dyslipidemia ↓ Hyperglycemia	(46)
Cardamom oil	3 g/kg, 8 weeks, p.o.	*In* *vivo,* Wistar rats	↓ TC ↓ LDL-C ↓ TG	(47)
1,8-cineole	10 μg/ml	*In* *vitro*	↓ HDL ↓ Lipid accumulation	(48)

TC: total cholesterol, TG: triglyceride, LDL-C: Low-density lipoproteins-cholesterol, T2DM: type 2 diabetes mellitus, HbA1C: hemoglobin A1c, HOMA-IR: homeostasis model assessment index, SIRT1: sirtuin-1, hs-CRP: high-sensitivity C-reactive protein, MDA: Malondialdehyde, HDL: high-density lipoprotein

**Table 2:** Therapeutic effects of cardamom and its active constituents on obesity

**Compounds**	**Dose**	**Study design**	**Results**	**Ref**
Cardamom	two 500 mg capsules, three times a day, 3 months	Clinical trial*, * overweight or obese patients with non-alcoholic fatty liver disease	↑ SIRT1 ↓ hs-CRP ↓ TNF-α ↓ IL-6 ↓ ALT ↓ Degree of fatty liver	(66)
Cardamom	3 g/day , 16 weeks	Clinical trial, obese women with polycystic ovary syndrome	↓ Anthropometric indices ↑ Glycemic indices ↓ Expression level of Carnitine palmitoyltransferase 1A, leptin receptor, and lamin A/C ↑ PPAR-γ	(67)
Cardamom	3g/day, 8 weeks	*In vivo*, Male Wistar rats	↓ Glucose intolerance ↓ abdominal fat deposition ↓ Dyslipidemia ↑ Antioxidant enzymes ↓ ALT, AST and ALP ↓ Fibrosis in liver	(16)

SIRT1: sirtuin-1, hs-CRP: high-sensitivity C-reactive protein, TNF-α: Tumor necrosis factor-alpha, ↓ IL-6: Interleukine-6, ALT: alanine aminotransferase, PPAR-γ: peroxisome proliferator-activated receptor gamma, AST: aspartate transaminase, ALP: alkaline phosphatase

**Table 3:** Therapeutic effects of cardamom and its active constituents on hypertension

**Compounds**	**Dose**	**Study design**	**Results**	**Ref**
Cardamom	3g/day, 12 weeks	Clinical trial*, * individuals with stage 1 hypertension	↓ Systolic, diastolic and mean blood pressure ↑ Fibrinolytic activity ↑ Antioxidant status	(68)
Cardamom	3g/day, 2 months	Clinical trial*, * pre-diabetic women	↓ TC ↓ LDL-C ↓ Insulin sensitivity	(43)
Cardamom	3-100 mg/kg	*In vivo*, rats	↓ Arterial blood pressure ↓ Phenylephrine (1 microM)-induced contractions	(69)
α-terpineol	25, 50, or 100 mg/kg/day, 1 week, p.o.	*In vivo*, rats	↓ Mean arterial pressure ↓ Vascular resistance ↑ Antioxidant status	(70)
α-terpineol	1, 5, 10, 20 and 30 mg/kg, i.v.	*In* *vivo,* Wistar rats	↓ Blood pressure ↑ Vasorelaxation	(71)
1,8-cineole	0.3-10 mg/kg, i.v.	*In vivo*, rats	↓ Mean arterial pressure ↓ Heart rate	(72)

TC: total cholesterol, LDL-C: Low-density lipoproteins-cholesterol

**Table 4:** Therapeutic effects of cardamom and its active constituents on diabetes

**Compounds**	**Dose**	**Study design**	**Results**	**Ref**
Cardamom	3g/day, 10 weeks	Clinical trial, T2DM patients	↓ HbA1C ↓ insulin ↓ HOMA-IR ↓ TG ↑ SIRT1	(44)
Supercritical carbon dioxide extract of cardamom seeds	550 mg/kg, 35 days, p.o.	*In vivo*, Wistar albino rats	↓ Fasting blood sugar	(81)
Supercritical carbon dioxide extract of cardamom seeds		*in vitro*	↑ Insulin sensitivity ↑Glucose uptake in the gut	(83)
Aqueous and methanol cardamom extracts	1 mg/mL	*in vitro*	↓ a-glucosidase activity ↓ a-amylase activity	(35)

HbA1C: hemoglobin A1c, HOMA-IR: homeostasis model assessment index, TG: triglyceride, SIRT1: sirtuin-1

## Conclusion

This manuscript reviewed the chief aspects of MetS and protective mechanisms of *E. cardamomum* and its active constituents in ameliorating and attenuating the components of MetS including dyslipidemia (by increasing the rate of cholesterol degradation, activating lipoprotein lipase, reducing lipid absorption from the intestine), obesity (through increasing the amount of SIRT1 and controlling some genes involved in obesity), hypertension (via blocking calcium channels, induction of cholinergic effects and anti-oxidant property), and high blood glucose (by enhancing the amount of SIRT1, PGC-1α, reducing NF-κB function and inhibiting α-glucosidase and α-amylase ) that have been reported in clinical trials, *in vivo*, and *in vitro* documents. Although there are growing numbers of animal studies demonstrating positive effects of cardamom in MetS, the number of studies linked to cardamom protective properties in humans is still limited. This review article also suggests that cardamom may be implicated as a preventative or therapeutic drug against MetS after being examined in different randomized clinical trials.

## Authors’ Contributions

HH and GK Study conception and design; RY Acquisition of data; RY Wrote the manuscript in consultation with MR and MGR; HH Critical revision.

## Conflicts of Interest

The authors express no conflict of interest.
